# Advanced Materials for Electrochemical Energy Conversion and Storage Devices

**DOI:** 10.3390/ma14247711

**Published:** 2021-12-14

**Authors:** Diogo M. F. Santos, Biljana Šljukić

**Affiliations:** 1 Center of Physics and Engineering of Advanced Materials (CeFEMA), Instituto Superior Técnico, Universidade de Lisboa, 1049-001 Lisbon, Portugal; biljana.paunkovic@tecnico.ulisboa.pt; 2 Faculty of Physical Chemistry, University of Belgrade, Studentski Trg 12-16, 11158 Belgrade, Serbia

Electrochemical energy conversion and storage is attracting particular attention due to the drawbacks and limitations of existing fossil fuel-based technologies. Progress in electrochemical energy conversion/storage devices takes three directions: batteries, supercapacitors, and fuel cells. Batteries find wide applications in portable devices, including laptop computers, mobile phones and cameras. Supercapacitors can accept and deliver charge at a much faster rate than batteries, and for many charge/discharge cycles. Fuel cells provide efficient and clean continuous power generation for stationary and portable applications. These three technologies are considered to be clean and promise to overcome climate change problems caused by the intensive use of fossil fuels.

However, issues related to electrode efficiency, membrane costs, and electrolyte stability still often limit the widespread commercialisation of electrochemical energy conversion/storage devices. Namely, the choice of electrode materials, as well as the electrolyte composition, determines the crucial electrochemical device parameters, such as specific energy and power, cycle life and safety. Accordingly, it is essential to develop the existing and introduce new procedures for synthesising electrode materials for batteries, capacitors and fuel cells. Developing new, improved electrocatalytic materials for batteries, supercapacitors, and fuel cell electrode reactions is expected to significantly impact device performance and, consequently, their commercialisation.

The present special issue is focused on recent developments in electrocatalytic materials for energy storage and conversion devices. It brings the latest advances in the synthesis and characterisation of novel materials for electrochemical energy conversion and storage devices, including high-efficiency lithium-ion rechargeable batteries, supercapacitors, and alkaline water electrolysers.

Lithium-ion batteries are the primary energy storage devices in the communications and renewable-energy sectors due to their high energy densities and lightness. In addition, they have no memory effect and do not use poisonous metals, such as lead, mercury or cadmium. Still, further enhancements are necessary concerning battery power, cycle life, safety, and cost, to meet some applications requirements. The performance of batteries strongly depends on the three main parts: the electrodes (anode and cathode) and the electrolyte solution properties. Thus, significant improvement in the electrochemical properties of electrode materials is essential to meet the demanding requirements of various battery applications.

A lithium-ion battery commonly comprises a carbon anode, a metal oxide (a layered oxide, a polyanion, or a spinel) cathode and a lithium salt in an organic solvent as the electrolyte. A separator in the form of a thin sheet of micro-perforated plastic is commonly employed to separate the anodic and cathodic parts while allowing ions to pass through. This special issue presents several novel cathode materials for LIBs. Thus, Hussmes et al. prepared a series of LiNi_1/3_Mn_1/3_Co_1/3_O_2_ cathode materials (doped with Al, Mg, Fe, and Zn) using a rather facile sol-gel method assisted by ethylenediaminetetraacetic acid (EDTA) as a chelating agent [1]. X-ray diffraction (XRD) analysis revealed that the degree of cation mixing, as well as the strain field, depended on the doping element. Their morphology inspected by scanning and transmission electron microscopy (SEM/TEM) was regular with hexagonal nanoparticles of practically the same size allowing proper assessment and comparison of their electrochemical performance. Doping with Mg and Al resulted in enhanced electrochemical performance as evidenced by the enhanced specific capacity, cyclability, and rate capability, owing to the minimum occupancy of foreign ions in the Li plane. Specifically, doping with Al increased the initial capacity (i.e., delivered higher discharge capacity in the first cycles compared to the Mg doping), but doping with Mg improved the long-term cyclability. Initial specific discharge capacity at 0.1 C decreased in the order Al-doped oxide (160 mAhg^−1^) > Mg-doped and undoped (ca. 150 mAhg^−1^) > Fe-doped (146 mAh g^−1^) > Zn-doped material (118 mAhg^−1^); doping with Fe and Zn had a negative effect on the specific discharge capacity value. On the other hand, specific capacity retention decreased in the order Mg-doped oxide (91%) > undoped (85%) > Al-doped (82%) > Fe-doped (67%) > Zn doped material (36%). The improved capability was attributed to the minimised local distortions in the lattice.

The work of Ajpi et al. presents the synthesis of lithium iron phosphate–polyaniline (LiFePO_4_–PANI) hybrid materials and their electrochemical performance as electrode materials for LIBs [2]. A high degree of crystallinity PANI was synthesised and optimised by chemical oxidation using ammonium persulfate and phosphoric acid. SEM analysis showed PANI formation with primary particles of 0.31 µm size and globular morphology with agglomerates of 2.75 µm. LiFePO_4_–PANI hybrid was subsequently synthesised by thermal treatment of LiFePO_4_ particles in a furnace with PANI and lithium acetate-coated particles under inert (Ar/H_2_) atmosphere. The morphologies of LiFePO_4_ particles and LiFePO_4_–PANI hybrid were observed to be similar, whereas their structures were found to be different due to the presence of PANI coating on the LiFePO_4_ particles. Elemental mapping confirmed a homogeneous distribution on the surface of the LiFePO_4_ particles.

The presence of PANI in the hybrid material was shown to have a beneficial effect on the hybrids’ electrochemical performance, i.e., on its rate capability. Namely, hybrid material with 25 wt.% PANI exhibited enhanced electrochemical behaviour in terms of capacity, rate capability and cyclability compared to the individual components, LiFePO_4_ and PANI. Thus, capacity at a charge/discharge rate of 0.1 C increased in the order PANI (95 mAhg^−1^) < LiFePO_4_ (120 mAhg^−1^) < LiFePO_4_–PANI (145 mAhg^−1^). The beneficial effect of PANI presence is also evident at a higher charge/discharge rate of 2 C, with a capacity of 70 and 100 mAhg^−1^ for LiFePO_4_ and LiFePO_4_–PANI, respectively. PANI improved the electronic transfer between LiFePO_4_ particles and the contact between LiFePO_4_ particles and electrolyte during charge/discharge. It can further serve as Li^+^ ion insertion–extraction host contributing to the capacity. Finally, PANI can act as a binder network between the LiFePO_4_ particles and the surface of the current collector.

For fuel cells to be considered a clean technology, the hydrogen used in H_2_/O_2_ fuel cells must be produced by clean/green methods. Still, most of the H_2_ produced globally represents the so-called “grey” hydrogen, i.e., it is obtained by natural gas reforming. Green hydrogen can be generated by water electrolysis powered with electricity from renewable energy sources such as wind or solar energy. The main problem of H_2_ production by water electrolysis is its high price. This high cost comes from the high energy input, i.e., large overpotential needed to split the water molecule. The overpotential can be reduced by using suitable electrode materials, i.e., with high activity for the hydrogen (HER) and oxygen (OER) evolution reactions. Cysewska et al. explored a series of nanostructured Mn-Co-based films as electrode materials for oxygen evolution reaction [3]. These films were prepared using different synthesis conditions, which were correlated with the films’ physicochemical properties (e.g., structure, morphology) and, consequently, with their electrocatalytic activity. Mn-Co nanofilm was directly electrochemically deposited in a one-step process on nickel foam from the solution containing only metal nitrates (Mn(NO_3_)_2_·4H_2_O and Co(NO_3_)_2_·6H_2_O) and no additives. XRD and X-ray photoelectron spectroscopy (XPS) analyses revealed that the as-prepared films consist mainly of Mn^2+^, Co^2+^, and Co^3+^ oxides/hydroxides and/or oxyhydroxides. The subsequent alkaline treatment of the film in 1 M KOH led to partial oxidation of Co^2+^ to Co^3+^ and generation of Mn^3+^, resulting in Mn-Co oxyhydroxides. The creation of crystalline Co(OH)_2_ with a hexagonal platelet-like shape structure was also observed by XRD and TEM analyses. Thus, the film’s final form is believed to have a layered double hydroxides structure, which has a highly beneficial effect on OER activity. SEM analysis showed that the Mn-Co films morphology on nickel foam was characterised by an interconnected 3D nanoflake structure with high porosity.

The Mn-Co films showed promising activity for OER in an alkaline medium (1 M KOH). Their electrocatalytic activity depended on the Mn/Co concentration ratio in the deposition solution and the deposition charge. The film obtained for Mn/Co ratio of 2 mM/8 mM and deposition time limited by a charge of 200 mC showed optimum physicochemical features (e.g., a high specific surface area of 10.5 m^2^g^−1^) and optimum electrochemical performance for OER. Thus, it exhibited high electrochemical stability with a low overpotential deviation between 330 and 340 mV at 10 mAcm^−2^ during 70 h.

Supercapacitors are high-performance devices with a high rate of charging/discharging and stability, i.e., capable of effective energy utilisation. As such, they are finding applications in elevators, forklifts, trucks, and buses, i.e., applications demanding substantial amounts of energy to be stored and released in a short time. However, energy densities provided by current supercapacitors are still not high enough to meet all the demands of the modern markets, limiting their usage. Supercapacitors are categorised into three main groups: pseudo-capacitors, electrochemical double-layer capacitors (EDLCs) and hybrid capacitors. Pseudo-capacitors involve a fast Faradaic mechanism (e.g., intercalation or redox reaction), unlike EDLCs that do not include Faradaic processes but the accumulation of ions induced by the adsorption of charged species at the electrode/electrolyte interface.

Supercapacitors comprise two porous electrodes separated by an ionically conducting electrolyte. Different materials, including polymers, carbon and metal oxides, can be used in capacitor electrodes. This special issue presents novel capacitor materials, undoped and Li-ion doped NiO, prepared by Bhatt et al. using a simple microwave method [4]. This approach resulted in the formation of crystalline nanomaterials with a cubic structure. X-ray diffraction analysis also revealed crystallite size decrease upon doping with Li-ion dopant. Furthermore, band gap decrease (3.3 eV for NiO vs. 3.17 eV for NiO doped with 1% Li) and an ultraviolet-blue emission along with a small amount of green emission were observed upon doping revealing the possibility of tuning the nanostructured NiO photoluminescence by doping. The optical results were confirmed and complemented by computational modelling. When it comes to electrochemical behaviour, the maximum reversibility during cyclic voltammetry studies was obtained for a NiO sample with 1% Li doping. The same sample further showed improved electrochemical properties, i.e., conductivity with a reduced charge transfer resistance of 5.592 × 10^−8^ Ω measured by electrochemical impedance spectroscopy, suggesting its potential application in supercapacitors.

Nofal et al. focused their research on electrolytes for supercapacitor applications, specifically on biopolymer-based electrolyte systems comprising methylcellulose (MC) as host polymer material and potassium iodide (KI) as the ionic source [5]. The electrolyte was prepared by a solution cast method using different amounts of KI. The complexation between MC polymer and KI salt was evident from Fourier-transformed infrared spectroscopy analysis. Electrochemical studies were carried out to identify the electrolyte with the highest conductivity for electrochemical double-layer capacitor applications. Increasing the KI concentration from 10 wt.% to 40 wt.% led to an increase of the charge carrier density and, consequently, to a decrease of the resistance (R_b_) of the charge transfer at the bulk electrolyte by three orders of magnitude (i.e., from 3.3 × 10^5^ to 8 × 10^2^ Ω) as determined by the electrochemical impedance spectroscopy measurements. The ionic conductivity was determined to be 1.93 × 10^−5^ Scm^−1^ and dielectric analysis confirmed the conductivity trends. A transference number of 0.88 indicated ions as the dominant charge carriers in the MC-KI electrolyte. The most conducting sample exhibited a wide electrochemical stability window up to 1.8 V during linear scan voltammetry study, pointing out its suitability as an electrolyte for EDLC application. Cyclic voltammetry with activated carbon electrodes displayed an absence of redox peaks and indicated the presence of a charged double-layer between the electrodes’ surface and electrolyte. A relatively high value of maximum specific capacitance, C_s_, of 113.4 F g^−1^ was determined at a polarisation rate of 10 mV s^−1^.

The guest editors believe that this special issue brings valuable guidelines for researchers in the area of electrochemical energy conversion/storage. Finally, we would like to express our gratitude to all authors for their valuable contributions to this special issue, as well as to all reviewers for their time and help in further improving the submitted papers.
